# Reversal of the neurological deficit in acute stroke with the signal of efficacy trial of auto-BPAP to limit damage from suspected sleep apnea (Reverse-STEAL): study protocol for a randomized controlled trial

**DOI:** 10.1186/1745-6215-14-252

**Published:** 2013-08-13

**Authors:** Jessica Kepplinger, Kristian Barlinn, Stanislava Kolieskova, Reza Bavarsad Shahripour, Lars-Peder Pallesen, Wiebke Schrempf, Xina Graehlert, Uta Schwanebeck, April Sisson, Charlotte Zerna, Volker Puetz, Heinz Reichmann, Karen C Albright, Anne W Alexandrov, Milan Vosko, Robert Mikulik, Ulf Bodechtel, Andrei V Alexandrov

**Affiliations:** 1 Department of Neurology, University of Technology Dresden, Fetscherstrasse 74, Dresden 01307, Germany; 2 Comprehensive Stroke Center, University of Alabama Hospital, 1813 Sixth Avenue South, RWUH M226, Birmingham, AL 35249, USA; 3 International Clinical Research Center, St. Anne’s University Hospital Brno, Pekarska 53, Brno, 656 91, Czech Republic; 4 Coordination Center for Clinical Studies, University of Technology Dresden, Fetscherstrasse 74, Dresden 01307, Germany; 5 Department of Neurology, General Hospital Linz (AKH), Krankenhausstrasse 9, Linz 4021, Austria

**Keywords:** Stroke, Sleep apnea, Non-invasive ventilatory treatment, BPAP, Early neurological deterioration

## Abstract

**Background:**

Although the negative impact of sleep apnea on the clinical course of acute ischemic stroke (AIS) is well known, data regarding non-invasive ventilation in acute patients are scarce. Several studies have shown its tolerability and safety, yet no controlled randomized sequential phase studies exist that aim to establish the efficacy of early non-invasive ventilation in AIS patients.

**Methods/design:**

We decided to examine our hypothesis that early non-invasive ventilation with auto-titrating bilevel positive airway pressure (auto-BPAP) positively affects short-term clinical outcomes in AIS patients. We perform a multicenter, prospective, randomized, controlled, third rater- blinded, parallel-group trial. Patients with AIS with proximal arterial obstruction and clinically suspected sleep apnea will be randomized to standard stroke care alone or standard stroke care plus auto-BPAP. Auto-BPAP will be initiated within 24 hours of stroke onset and performed for a maximum of 48 hours during diurnal and nocturnal sleep. Patients will undergo unattended cardiorespiratory polygraphy between days three and five to assess sleep apnea. Our primary endpoint will be any early neurological improvement on the NIHSS at 72 hours from randomization. Safety, tolerability, short-term and three-months functional outcomes will be assessed as secondary endpoints by un-blinded and blinded observers respectively.

**Discussion:**

We expect that this study will advance our understanding of how early treatment with non-invasive ventilation can counterbalance, or possibly reverse, the deleterious effects of sleep apnea in the acute phase of ischemic stroke. The study will provide preliminary data to power a subsequent phase III study.

**Trial registration:**

Clinicaltrials.gov Identifier: NCT01812993

## Background

Stroke still constitutes a leading cause of death and ranks number one among all causes of serious long-term adult disability in Europe and the United States [1,2], underscoring the need for adjuvant acute and preventive stroke therapies that reduce both the devastating human and economic burden of this disease.

More than two-thirds of acute ischemic stroke (AIS) patients suffer from sleep apnea of variable degree compared to 5 to 15% in the general population [3-6]. Moreover, in a prospective cohort study, patients with moderate to severe obstructive sleep apnea (OSA) had an almost two-fold increased risk for stroke and death after adjustment for other traditional vascular risk factors, thus establishing OSA as an independent risk factor [7,8]. Following stroke, sleep apnea negatively affects the length of hospitalization and short-term as well as long-term outcomes, likely through its association with an increased risk of neurological deterioration during acute and subacute phases of stroke [4,9-12]. Neurological deterioration during the early phase of AIS can occur between one and two out of five patients and it is independently predicted by sleep apnea and associated with unfavorable outcome [9,13].

Numerous studies including serial transcranial Doppler (TCD) in patients with AIS provided evidence that persisting large-artery steno-occlusive disease and corresponding collateral failure, leading to depletion of oxygen and nutrients to penumbral tissues, appear to be the most relevant pathogenic mechanisms for neurological deterioration [14-17]. Thus, it seems intuitive that further hemodynamic and perfusion derangements may do harm by facilitating hypoperfusion and ultimately lead to expansion of infarction to penumbral areas (or even oligemic tissues surrounding the ischemic penumbra) with neurological deterioration as the clinical correlate [18].

Several pathophysiological conditions associated with sleep apnea are believed to account for acute harmful effects on ischemic brain [19]. Recurrent nocturnal apneic episodes promptly entail intermittent hypoxemia, intrathoracic pressure changes and activation of the sympathetic nervous system resulting in cardiac arrhythmias and marked blood pressure swings [20,21]. The cerebral autoregulatory system is essential to compensate for changes in systemic hemodynamics and to avoid harmful hypo- or hyperperfusion of brain tissue. The fate of ischemic brain tissues particularly in the setting of large steno-occlusive disease already depend on dilated arteries with no further or only minimal residual vasomotor capacity [22-24] to counteract apnea-associated blood pressure changes and maintain blood flow according to local metabolic needs [19]. In fact, patients with sleep apnea generally have impaired cerebral vasoreactivity, even during wakefulness, potentially explaining their particular vulnerability to stroke-associated hemodynamic derangements [25-28]. Repetitive oxygen desaturations in cerebral ischemic tissue during apneic periods may further compromise functional and structural integrity of neuronal cells [29]. Additionally, hypercapnia may lead to depletion of the collateral blood flow when vessels in the non-ischemic area dilate more leading to blood flow diversion from the ischemic area to the non-ischemic areas, possibly further worsening hypoperfusion in the ischemic brain area [23,30,31]. This ‘cerebral blood flow steal’ phenomenon was demonstrated by TCD in real time in AIS patients during voluntary breath holding and led to early neurological deterioration in those with acute proximal arterial obstructions and excessive daytime sleepiness [10]. When found during the initial hospitalization for AIS, this so called ‘reversed Robin Hood syndrome’ led to a four-fold increase in stroke recurrence within the same arterial territory [11].

Maintenance of stable systemic and cerebral hemodynamics, as well as adequate and constant brain tissue oxygenation, plays a crucial role in the management of AIS [32]. The ischemic penumbra may be present for many hours and be amenable to reperfusion. Consequently, any treatment that potentially maintains or augments cerebral perfusion may be justified [33]. Non-invasive ventilation with either continuous (CPAP) or bilevel positive airway pressure (BPAP) is the treatment of choice for sleep apnea. Although some studies have shown the safety and beneficial effects of CPAP and BPAP by improvement of quality of life and reduction of cardio- and cerebrovascular morbidity and mortality in the general sleep apnea population [34,35], their role in AIS patients remains to be elucidated. Most previous investigations were conducted beyond the (hyper-) acute phase of ischemic stroke and pursued secondary stroke prevention goals rather than acute effects. For example, two studies confirmed safety and tolerability, and pointed to a potential short-term treatment effect of prompt initiation of non-invasive ventilation in the acute phase of stroke when hemodynamically compromised tissue can potentially be salvaged [36,37]. In the first study, an observational study, AIS patients with proximal persisting arterial occlusions and suspected or known OSA had a tendency towards early neurological improvement (median National Institutes of Health Stroke Scale (NIHSS) score decrease by 2 points) during the hospital course when BPAP was initiated within the first 24 hours of admission, as compared with those who did not receive BPAP (median NIHSS score decrease by 1 point, *P* = 0.078) [36]. The other study randomly assigned fifty patients in the first night after AIS to either CPAP for three nights or control [37]. The investigators found that CPAP treatment was feasible, safe and associated with a greater neurological improvement until day eight in patients who tolerated CPAP well when compared with control patients (NIHSS 2.3 versus 1.4, *P* = 0.022).

As data on the use of non-invasive ventilation in the acute phase of ischemic stroke are lacking and its potential benefit on stroke outcomes is controversial [38], a randomized controlled efficacy trial appears justified as being timely and needed. The choice of using an auto-titrating BPAP ventilation mode is based on the fact that AIS patients will not have the necessary time or access to facilities to undergo comprehensive sleep laboratory testing prior to start of treatment. Auto-BPAP adapts pressure settings automatically according to apnea frequency recorded in real-time from breath-to-breath, thus sparing manual titration of therapeutic pressures. Also, BPAP mode delivers pressure levels for inspiration and expiration separately, easing inspiration while ensuring alveolar ventilation and splinting the airway, which has several advantages over standard CPAP that applies a fixed pressure setting during the entire respiratory cycle [39]. Collectively, auto-BPAP may be superior particularly in patients who require higher pressure levels [39,40] and produces improved patient adherence, a well-known issue in recent studies that may be even more important for stroke patients who may not have appropriate understanding and motivation to allow themselves to be exposed to mask and airflow pressures. Finally, this approach could be utilized in stroke centers without experience in sleep medicine.

## Methods

### Study design

This is an international, multicenter, prospective, randomized, controlled, third rater-blinded, parallel-group phase II study (Clinicaltrials.gov identifier: NCT01812993). A double-blind study design would require sham ventilatory treatment that is impossible in this setting. A sham mask device with subtherapeutic pressures may also pose a risk of harm as it will likely preclude adequate ventilation and increase the respiratory ‘dead space’ volume. Thus, to minimize bias associated with an open-label study, a trained neurologist or nurse blinded to treatment allocation will assess the primary and, whenever applicable, secondary efficacy outcomes. The study protocol has received full ethics approval by the Institutional Review Board of the University of Technology Dresden (Reference number: EK 362112012).

### Patient population

Using a convenience sampling method, patients presenting to one of the participating stroke centers with AIS due to intra- or extracranial arterial steno-occlusive obstruction and clinically suspected sleep apnea will be eligible for the study. Patients will have to meet the following inclusion and exclusion criteria:

#### Inclusion criteria

•Male and female patients 18 to 80 years;

•Clinical suspicion of an AIS (measurable or fluctuating neurological deficit with a NIHSS ≥ 4 points) within 24 hours from symptom onset;

•Extracranial (that is, internal carotid artery) or intracranial (that is, internal carotid artery; middle/anterior/posterior cerebral arteries) ≥ 50% stenosis, near-occlusion or occlusion diagnosed by ultrasound, computed tomography angiography (CTA) or magnetic resonance angiography (MRA), corresponding to the acute neurological deficit;

•High risk of having sleep apnea (classified by the Berlin sleep apnea questionnaire); or history of known sleep apnea; or witnessed repetitive apneic episodes during sleep or somnolence during hospitalization;

•Written informed consent by participants; alternatively by proxy or two physicians when not obtainable by patient or proxy (according to local regulations).

#### Exclusion criteria

•Perceived course towards malignant middle cerebral artery infarction;

•Vertebrobasilar ischemic stroke;

•Immediate or perceived need for intubation;

•Known sleep apnea currently on non-invasive ventilatory treatment;

•Standard contraindications for non-invasive ventilatory treatment;

•Pre-morbid modified Rankin scale (mRS) score ≥ 3;

•Severe comorbidities (that is, severe heart failure, severe obstructive lung disease, active malignant disease, severe dementia);

•Pregnant and breast-feeding women;

•Participation in another clinical trial other than standard-of-care registry.

### Randomization

All randomization procedures are prepared and monitored by the Dresden Coordination Center for Clinical Studies, independent of patient recruitment and implementation of the assignment. After eligibility for the study has been confirmed, written informed consent obtained and baseline assessments completed, a 1:1 randomized allocation to either standard stroke care alone (no ventilatory treatment = control) or standard stroke care plus non-invasive ventilatory treatment with auto-BPAP will be performed according to a computer-generated random number (nQuery Advisor® PPS 6.01, Saugus, MA, USA) that is provided via the online Research Electronic Data Capture (REDCap) system [41]. The allocation sequences are concealed from investigators involved in patient recruitment and collection of outcome data. Stratified block randomization with strata for study site and stroke severity as defined by the NIHSS (that is, 4 to 10 and > 10 points) will be used, wherein the block size is fixed and will not be communicated to any of the investigators with clinical involvement in this trial.

### Study procedures

**Figure 1:**
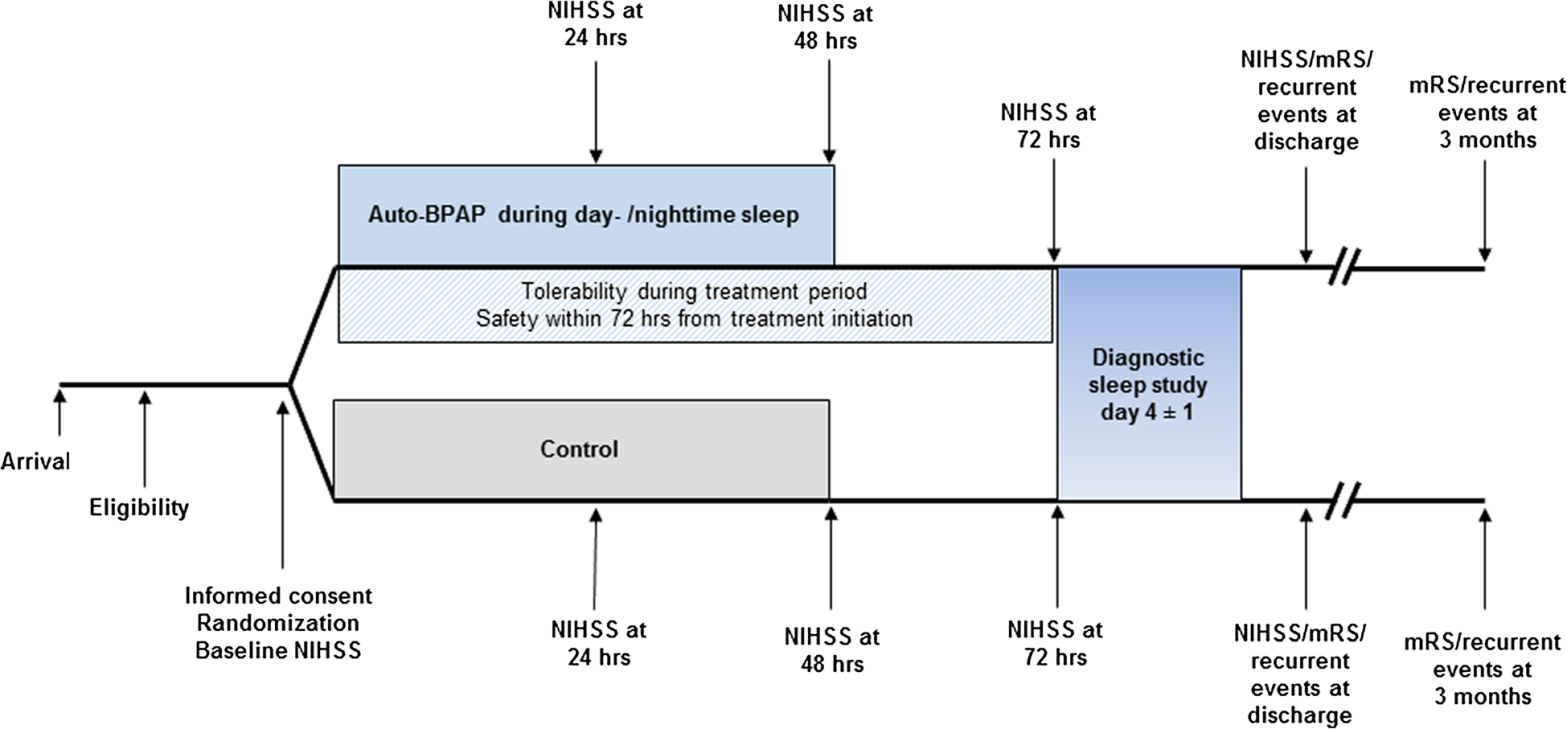
**Study flowchart.** NIHSS indicates National Institutes of Health Stroke Scale Score. BPAP, bilevel positive airway pressure. mRS, modified Rankin Scale.

Study physicians will obtain baseline NIHSS scores, determine patient eligibility and obtain informed written consent from each patient prior to any study-related procedures. Patients allocated to the active group will receive non-invasive ventilatory treatment with auto-BPAP (Philips Respironics, Auto-BIPAP Biflex®, Herrsching, Germany) within 24 hours of symptom onset, and for a maximum of 48 hours during diurnal and nocturnal sleep or somnolence, from the beginning of study randomization (Figure 1). This approach warrants that all patients in the active group will receive an intention-to-treat all potential apneic episodes independently of their sleep cycle and somnolence. During the study period, oral and tube feeding intake will be allowed according to local institutional protocols. The auto-BPAP device automatically adjusts expiratory (EPAP) and inspiratory positive airway pressure (IPAP) levels to meet patients’ needs and will be configured to pressure settings that are identical in all patients in the active group: possible minimum EPAP 4 mbar; possible maximum IPAP 25 mbar; maximum difference between EPAP and IPAP (delta) 8 mbar; Biflex 2. The device records sleep-related respiratory events as well as duration of its use. Attachment of the device and initiation of treatment is performed by the study personnel who will receive training specific to the device by a sleep neurologist experienced with the study device.

All patients will undergo a diagnostic unattended cardiorespiratory polygraphy between days three and five from enrollment for assessment of sleep apnea using a previously established protocol [5]. A rater experienced in sleep medicine and who is unaware of group allocation and clinical data will analyze the sleep polygram.

### Primary aim

The primary aim is to determine if early auto-BPAP initiated within the acute phase of ischemic stroke affects short-term clinical outcome. The signal-of-efficacy in these patients will be determined if these early changes in the neurological deficit could be sustained at three months from symptom onset.

### Primary endpoint

Early neurological recovery will be assessed as any improvement on the NIHSS score at 72 ± 12 hours from randomization. Physicians or qualified study personnel, other than those involved in acute treatment (that is, blinded to randomization) and who are certified in performing NIHSS scoring, will obtain the NIHSS scores.

### Secondary aims

Secondary aims are comprised of the assessment of tolerability and safety of auto-BPAP initiated within the acute phase of ischemic stroke, as well as its possible effect on other clinical outcomes during hospitalization and follow-up (signal-of-efficacy).

### Secondary endpoints

Tolerability will be assessed by patient adherence to auto-BPAP (defined as tolerating the treatment during sleep or somnolence for at least four hours continuously).

*Safety* will be assessed by:

(i) frequency of serious adverse events (that is, aspiration, aspiration pneumonia defined as combined radiologic, white blood count and clinical findings, respiratory failure with/without intubation) during treatment period that in the opinion of the study physician are causatively and timely (for a maximum of 72 hours from treatment initiation) related to auto-BPAP. For comparison, patients in the control group will be monitored for respiratory complications within 72 hours from randomization. All deaths in both groups during hospital stay;

(ii) frequency of all complaints and possible side effects of auto-BPAP (that is, local irritation of skin/mucosa, mucosal dryness, nausea/vomiting);

(iii) any concerns by hospital nursing staff will be documented as adverse events since patients will be under standard of care repeated assessments set by admission protocols and treating physicians.

Signal-of-efficacy:

Clinical and functional outcomes will be assessed by:

(i) frequency of neurological deterioration (increase in baseline NIHSS score ≥ 4 points) at 24, 48 and after 72 hours from randomization by blinded observers;

(ii) frequency of early neurological improvement (decrease in baseline NIHSS score ≥ 4 points) at 24, 48 and after 72 hours from randomization by blinded observers;

(iii) good functional outcome (mRS score 0 to 2) at discharge and at three months by blinded observers, and ordinal analysis of the mRS at three months;

(iv) all deaths and any TIA or new ischemic stroke during hospitalization or within three months of protocol initiation.

### Data Safety Monitoring Board (DSMB)

Serious adverse events (SAE) as described above and unexpected SAE will be collected for the first 72 hours from initiation of auto-BPAP. All SAEs will be reported by fax or Email within 24 hours from occurrence to the DSMB that is composed of two clinicians and a biostatistician at the University of Technology Dresden who is not involved in the execution of this study. In the event of an SAE, subjects in the auto-BPAP arm will immediately have the procedure terminated, and will receive stabilizing procedures. All SAEs must be reported within 24 hours, and will require submission of incident findings and associated events by the local principal investigator to the DSMB for adjudication. From our pilot study [36], we expect SAEs to occur in less than one in ten enrolled patients. If there is one SAE in the first 10 patients, the study will continue. If there are two SAEs in the first 10 patients then the study will stopped and DSMB will make recommendations if certain criteria/exposures need to be modified. The study will be halted if treatment with auto-BPAP results in more than two SAEs per 10 patients. Thereafter, DSMB will receive monthly enrollment/safety/outcomes reports. Additionally, DSMB will be convened any time that safety or data concerns arise. DSMB will also have access to unblinded data, to make decisions if higher than expected rates of SAEs are being outweighed by potential benefits; no further criteria for stopping the trial will be set as limited data are available to guide such algorithms at this point.

### Sample size

Sample size estimates were calculated from a previous randomized feasibility study [37], where a significantly greater improvement in the NIHSS score was observed when AIS patients were treated with CPAP as compared with controls (mean 2.3 versus 1.4 points, *P* = 0.022). This minimal difference of 0.9 points in NIHSS improvement between the groups was considered clinically relevant. Assuming a two-sided 5% significance level and 80% power, 24 patients in each group (48 patients in total) would be needed to detect such a difference in the mean NIHSS score at 72 hours (nQuery Advisor ® 6.01, Saugus, MA, USA). In order to reflect missing data and possible issues with tolerability (for example, loss to follow-up, lack of patient compliance) at a maximum rate of 20%, the sample size for this study is set at 60 patients (30 per group).

To minimize missing data in this trial, all outcome assessments will be electronically scheduled for each enrolled patient and reminders will be electronically generated and delivered to the assessors on time. Also, to prevent drop-outs due to non-compliance with auto-BPAP in the active treatment arm, patients will be closely monitored by a carefully trained nurse to enhance tolerability of the auto-BPAP device. After discharge, all patients will be contacted and interviewed either face-to-face or by telephone. In patients who are not able to communicate, proxies will be involved.

### Planned statistical analysis

Research Electronic Data Capture (REDCap) [41] will be used for recruitment and data management in this study. The primary analysis will be an intention-to-treat analysis in which all subjects are retained in the group to which they were allocated and included in the analysis, irrespective of their compliance with allocated treatment. Statistical analysis in this population is used to determine statistical significance. The per-protocol population with inclusion of all patients without major protocol violations will be also analyzed. For confirmatory analysis, the primary endpoint will be assessed using the Mann-Whitney *U-*test. For secondary endpoints, categorical variables will be assessed using Chi-square tests or Fisher’s exact test, while continuous variables will be assessed using Student’s *t*-test and the Wilcoxon rank sum test, where appropriate. With regard to the exploratory analysis, adjustments will not be made for multiple comparisons. Multivariate analysis will be performed to define positive and negative predictors of specific primary and secondary outcomes. Last-observation-carried-forward method will be used to impute missing data. Statistical significance will be declared if the two-tailed *P*-value will be less than 0.05. Two-sided 95% confidence intervals will be calculated for continuous variables. All statistical analyses will be performed with SPSS (SPSS Inc., Chicago, IL, USA) and SAS (SAS Institute, Inc., Cary, NC, USA) software.

## Discussion

We expect that this study will advance our understanding of how early treatment with non-invasive ventilatory treatment can counterbalance, or possibly reverse, the deleterious effects of sleep apnea in the acute phase of ischemic stroke. Our assumptions for sample size were based on a relatively small effect size as even slight improvements on the NIHSS score appear to be a strong predictor of good functional outcome after stroke [42]. The study will provide preliminary data to power a phase III study of auto-BPAP in the acute phase of cerebral ischemia.

## Trial status

At the time of manuscript submission, recruitment of study patients is ongoing. Initial recruitment was started in May 2013.

## Abbreviations

AIS: Acute ischemic stroke; BPAP: Bilevel positive airway pressure; CPAP: Continuous positive airway pressure; CTA: Computed tomography angiography; DSMB: Data safety monitoring board; EPAP: Expiratory positive airway pressure; IPAP: Inspiratory positive airway pressure; MRA: Magnetic resonance angiography; mRS: Modified Rankin scale; NCT: National clinical trial; NIHSS: National Institutes of Health Stroke Scale; OSA: Obstructive sleep apnea; REDCap: Research electronic data capture; SAE: Serious adverse events; TCD: Transcranial doppler.

## Competing interests

The authors declare that they have no competing interests with regard to this study.

## Authors’ contribution

Each author has made substantial contributions to conception and design, or acquisition of data, or analysis and interpretation of data. JK, KB, XG, US, AWA, UB, and AVA have contributed to the study design. XG, US, and KA were involved in statistical analyses. JK, KB, SK, RS, LPP, WS, AS, CZ, MV, and RM contributed to the implementation of the study. JK, KB, VP, HR, AWA, UB, and AVA were involved in drafting the manuscript and revising it critically for important intellectual content. All authors have given final approval of the version to be published.

## Authors’ information

Ulf Bodechtel and Andrei V Alexandrov are the joined senior authorship.
